# Location-Aware Node Management Solution for Multi-Radio Dual Connectivity Scenarios

**DOI:** 10.3390/s21227450

**Published:** 2021-11-09

**Authors:** Jesús Burgueño, Isabel de-la-Bandera, Raquel Barco

**Affiliations:** Instituto Universitario de Investigación en Telecomunicación (TELMA), Universidad de Málaga, CEI Andalucía TECH, 29010 Málaga, Spain; ibanderac@ic.uma.es (I.d.-l.-B.); rbm@ic.uma.es (R.B.)

**Keywords:** multi-radio dual connectivity, location-awareness, cellular network node management

## Abstract

The location of user equipments (UEs) allows application developers to customize the services for users to perceive an enhanced experience. In addition, this UE location enables network operators to develop location-aware solutions to optimize network resource management. Moreover, the combination of location-aware approaches and new network features introduced by 5G enables to further improve the network performance. In this sense, dual connectivity (DC) allows users to simultaneously communicate with two nodes. The basic strategy proposed by 3GPP to select these nodes is based only on the power received by the users. However, the network performance could be enhanced if an alternative methodology is proposed to make this decision. This paper proposes, instead of power-based selection, to choose the nodes that provide the highest quality of experience (QoE) to the user. With this purpose, the proposed system uses the UE location as well as multiple network metrics as inputs. A dense urban scenario is assumed to test the solution in a system-level simulation tool. The results show that the optimal selection varies depending on the UE location, as well as the increase in the QoE perceived by users of different services.

## 1. Introduction

The position tracking of mobile network users has enabled to develop a wide variety of new applications nowadays, from assisted driving services [1] to the customization of points of interest recommendation [2]. In these cases, users share their location to receive more advanced services. However, the network operator can also use the location of the user equipments (UEs) to enhance their condition within the mobile network, improving the quality of experience (QoE) perceived in the applications they use. For example, the user location can be used to detect and manage network failures by generating new indicators about areas of interest [3] or to decrease the interference level by adjusting the transmission power [4,5]. These features can increase the overall performance obtained in the mobile network, which can lead users to perceive a better QoE because the increased network availability or the higher average Signal-to-Interference-plus-Noise Ratio (SINR).

Moreover, the relevance of the location-awareness functionalities acquire a greater magnitude when combined with new fifth-generation (5G) technologies deployed on nowadays non-standalone networks [6]. For example, the authors of [7] use the location of users to noticeably decrease the interference level when beamforming is used. In a similar context, multi-radio dual connectivity (MR-DC) is one of the 5G features that allows more flexibility in network resource management on non-standalone networks. It allows UEs to simultaneously exchange data with one eNB (evolved Node B) and one gNB (5G New Radio node B), i.e., two nodes that belong to 4G and 5G, respectively [8]. Two different roles will be assigned to these nodes. One node is selected as master, which exchanges signalling between the UE and the core network (CN), as well as defining the radio resource control (RRC) state. In addition, the master node also sends data to the UE. On the other hand, the other node will be assigned as secondary role, only delivering data to the UE.

Among other benefits, MR-DC allows UEs to utilize more capacity to reach higher data rates or, alternatively, to send the same information through both links to increase the communication reliability. For instance, the simultaneous transmission of the same data packet through multiple links is addressed in [9]. In this way, it allows to improve the communication reliability as well as latency. Moreover, a mechanism that decides the transmissions to be duplicated is introduced in [10]. It further improves the spectral efficiency. On the other hand, the uneven allocation of resources of the serving nodes allows optimizing the network performance for different purposes. In this way, the authors of [11] propose that each node provides different flow of data in order to minimize the user energy consumption. Similarly, the scheme proposed in [12] pursues a compromise between the minimum energy consumption of the serving nodes and the highest possible Quality of Service (QoS) provided to the UE. The authors of [13] detail a load balancing approach in which the node serving a smaller number of users delivers more traffic. It manages to maintain the QoS required by the already established UEs while the number of UEs in the network increases. In turn, a scheme that aims at minimizing the delay of the complete transmission is introduced in [14]. For this purpose, lower traffic flow is delivered by the node that involves a greater delay.

In addition, an appropriate selection of the eNB and gNB that serve each UE allows to maximize this improvement achieved by managing the resources of the suitable nodes. In this regard, the authors of [15] calculate the combination of nodes that maximizes the sum rate capacity. It is computed by using the SINR reported to each node by the UE. However, this solution is proposed for scenarios with a low number of users and nodes since it uses a brute-force algorithm, so it can trigger the number of different combinations in denser environments. In a similar way, an iterative algorithm that selects two nodes, one from a macro-cell and one from a small-cell, is proposed in [16]. It also uses the SINR to maximize the throughput obtained by UEs. On the other hand, the authors of [17] propose to dynamically add or remove the nodes which simultaneously serve the UE based on Channel State Information (CSI) and cell loads. This scheme aims at increasing the data rate, as well as decreasing the probability of radio link failure. Including the use of the UE location, a deep learning-based mobility management methodology is introduced in [18] that enables dual connection to improve the quality of service of UEs that are going to perform a handover. To this end, the methodology forecasts the future location of users based on mobility patterns. Besides the selection of nodes from two 3GPP access technologies, other studies propose the use of a wireless local area network access point (WAP) to serve the UE in heterogeneous networks [19]. Therefore, both licensed and unlicensed spectrum is used in resource allocation. In this manner, the authors of [20] provide a methodology that dynamically balances the load between eNBs and WAPs. On the other hand, the nodes of these two radio access technologies (RATs) that provide the highest estimated QoE to video streaming users are selected by the approach introduced in [21].

In spite of these solutions, 3GPP proposes to select the nodes based only on Reference Signal Received Power (RSRP) [22]. Given the simplicity of this solution, this paper proposes an alternative methodology to outperform 3GPP approach. The proposed system uses the location of the UEs as well as multiple network metrics to select the eNB and gNB that will provide the maximum QoE to users. In this context, this paper proposes a location-aware solution for the selection of eNB and gNB that simultaneously serve an user in MR-DC-enabled scenarios. A system-level simulation tool is used to compare the proposed methodology and the 3GPP-compliant solution in a real dense urban scenario. In this regard, several tests are carried out with different scenario conditions to demonstrate the feasibility of the proposed solution, which enhance the overall QoE perceived by users. The results also demonstrate the importance of location in properly assigning network nodes in MR-DC scenarios.

The rest of the paper is organized as follows. Section 2 presents the scenario. The methodology proposed is detailed in Section 3 followed by the simulation assumptions in Section 4. The results are then depicted in Section 5. Finally, concluding remarks and future work are outlined in Section 6.

## 2. Scenario

Several services provided over cellular networks are increasingly demanding more bandwidth. To address this issue, in addition to the use of new features introduced in current non-standalone networks such as MR-DC or millimeter-waves, network operators are deploying network topologies with higher spectrum reuse [23]. Hence, the node inter-site distance and transmit power are decreased to set up denser scenarios, which allows to increase the reuse of the same frequencies. This topology is defined by 3GPP as dense urban scenarios, which proposes 200 m as average inter-site distance [24].

These scenarios can encompass many types of areas with different characteristics, such as user density, predominant services, obstacles distribution, eventuality of social events, etc. For example, residential areas will have the most indoor users, while rural areas will have a low density of users. Moreover, video services will be more frequently used in a residential zone than in a park. There will also be spots with high frequency of social or sporting events, etc. Therefore, in addition to network performance indicators, the characteristics of the environment surrounding the nodes and users must be considered to properly manage network resources.

Furthermore, the service of the UE must be taken into account to maximize his perceived QoE, since each service can have different requirements. This perceived QoE is usually measured using the mean opinion score (*MOS*), which ranges from 1 (poor experience) to 5 (excellent experience) [25,26]. Since the availability of QoE data reported by actual users is often scarce, the MOS is computed using utility functions mapping high-level service performance indicators, e.g., initial playback time for video streaming services [27]. Therefore, different QoS parameters, such as latency or reliability, are intrinsically involved in estimating users’ level of satisfaction depending on the type of service [28,29]. Hence, the UE location and a different QoE model for each service are used to maximize the MOS provided by the methodology introduced in the next Section 3.

## 3. Node Management Methodology

The proposed technique selects the eNB and gNB that will serve each user with the highest possible MOS in MR-DC-enabled scenarios. To this end, a set of indicators is used as input of the system. They are detailed as follows:

- **Service:** The service used by the UE. In this regard, the selection could vary depending on the requirements of the service, i.e., the importance of the throughput, communication reliability, delay, etc. to perceive a good QoE. Thus, the system will use a different configuration for each service.
- **Location**: The location of the UE. In this sense, the configuration of the system could also change depending on the characteristics of the area that surrounds the UE. In this study, the network operator is assumed to be aware of the UE position by trilateration [30]. This process computes the intersection between circles created between the UE and different network nodes taking advantage of the received signal strength.
- **Reference Signal Received Power (RSRP)**: The RSRP of each candidate eNB and gNB reported by the UE. Only the nodes that overcome a threshold will be proposed as candidates.
- **Reference Signal Received Quality (RSRQ)**: The RSRQ of each candidate eNB and gNB reported by the UE. It may not correlate with the RSRP in some cases, such as when both the received power and interference level are high.
- **Available Physical Resource Blocks (PRB)**: The number of free PRBs in each candidate eNB and gNB. It may rely on the density of users as well as the average bandwidth assigned to each user, which can also depend on the predominant service.

As introduced above, it is important to know the characteristics of the area that surrounds the user before assigning the network nodes. This methodology proposes to divide the scenario into M∗N squares, whose surface is assumed to have the same characteristics. The experiences of historical users of each square are used to forecast the MOS that can be provided by each node. With this objective, the RSRP and RSRQ reported, as well as the resource availability of the eNB and the gNB that simultaneously served these past users, are proposed to estimate the MOS. Therefore, as each metric could have a different weight to obtain a good QoE for different squares or services, M∗N∗S estimation models are generated from the introduced metrics to estimate the expected MOS for each service in each square, where *S* is the number of different services. For instance, the weight of the resource availability could be higher in ultra-dense areas. On the other hand, RSRQ could be more relevant for video users demanding high throughput, while RSRP could be more important for lightweight communications with Internet-of-Things (IoT) sensors. Hence the importance of having different estimation models. Once they are generated and deployed to assign the serving nodes in real time, the network operator will select the corresponding model based on user location and service. It will then take the measurements reported by the UE and the resource availability of each candidate eNB and gNB to assign the nodes that maximize the MOS and use them as inputs for the following algorithm.

To decrease the computing cost, only the nodes that provide a RSRP over a threshold will be considered as candidates. Moreover, a lightweight technique is used to generate the estimation models: multiple regression technique [31], which is detailed in Equation (1):

(1) $$\left[\begin{array}{c} QoE_{1}\\ QoE_{2}\\ \vdots \\ QoE_{n} \end{array}\right]=\left[\begin{array}{cccc} 1 & PRB_{1} & RSRP_{1} & RSRQ_{1}\\ 1 & PRB_{2} & RSRP_{2} & RSRQ_{2}\\ \vdots & \vdots & \vdots & \vdots \\ 1 & PRB_{n} & RSRP_{n} & RSRQ_{n} \end{array}\right]\left[\begin{array}{c} C\\ W_{Load}\\ W_{Power}\\ W_{Quality} \end{array}\right]+\left[\begin{array}{c} Error_{1}\\ Error_{2}\\ \vdots \\ Error_{n} \end{array}\right]$$

where *n* represents the number of historical samples of the same service in a single square from a dataset collected by testing the 3GPP solution in the same scenario. On the other hand, $QoE_{i}$ indicates the *MOS* estimated for the UE *i* by using network Key Quality Indicators (KQIs) as detailed in [32]. In addition, $PRB_{i}$, $RSRP_{i}$ and $RSRQ_{i}$ represent the number of free PRBs, the signal level and quality metrics, respectively. It should be pointed out that these three measurements represents the mean of the values corresponding to both eNB and gNB, as the MOS value is the result of the service provided by both nodes. Therefore, the estimation model consists of *C*, which is the constant value of the model, and the weights (*W*) of each introduced metric. In this regard, *C*, $W_{Load}$, $W_{Power}$ and $W_{Quality}$ are derived using the historical samples of QoE, PRB, RSRP and RSRQ, and the use of an ordinary least squares estimator. This process is detailed in [33]. Afterwards, the QoE will be estimated for future situations as indicated in Equation (2):

(2) $$E_{t}=C+W_{Load}*PRB+W_{Power}*RSRP+W_{Quality}*RSRQ$$

where $E_{t}$ is the MOS value estimated by the proposed approach. Therefore, the eNB and the gNB that provide the highest $E_{t}$ values will be selected to serve the UE.

In addition, the Mean Absolute Percentage Error (*MAPE*) metric is computed to know how reliable is the created model. It indicates the difference between the MOS value $R_{t}$ calculated using the QoE equation of the corresponding service introduced in [32] and the $E_{t}$ value estimated by the proposed methodology (Equation (3)),

(3) $$MAPE\left(\%\right)=\frac{100}{n}\sum_{t=1}^{n}\left|\frac{R_{t}-E_{t}}{R_{t}}\right|$$

**Figure 1:**
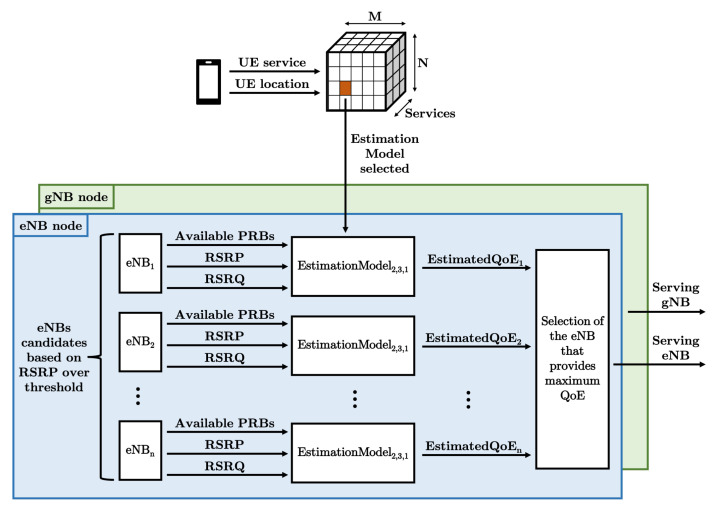
Block diagram of the proposed methodology.

The lower the *MAPE*, the higher the estimation accuracy. Therefore, the selection of nodes will be made with more reliability as the MAPE decreases, leading to further increase the overall QoE. The block diagram of this node management solution is represented in Figure 1. It should be noted that in this figure $EstimationModel_{2,3,1}$ is selected because the UE uses the first service and is located in the orange square, which corresponds to the second column and third row in this example. Finally, the algorithm of the proposed methodology is detailed in Algorithm 1.
**Algorithm 1** Node management solution1: [*C,W_Load_,W_Power_,W_Quality_*] ← Stored estimation model for the corresponding square and service2: **for** each eNB and gNB **do**3:  **if**
*RSRP_reported_* > *Threshold*
**then**4:   *E_t_* ← (2)5:  **end if**5: **end for**7: Serving eNB and gNB ← eNB and gNB providing the highest *E_t_*

## 4. Simulation Assumptions

**Figure 2:**
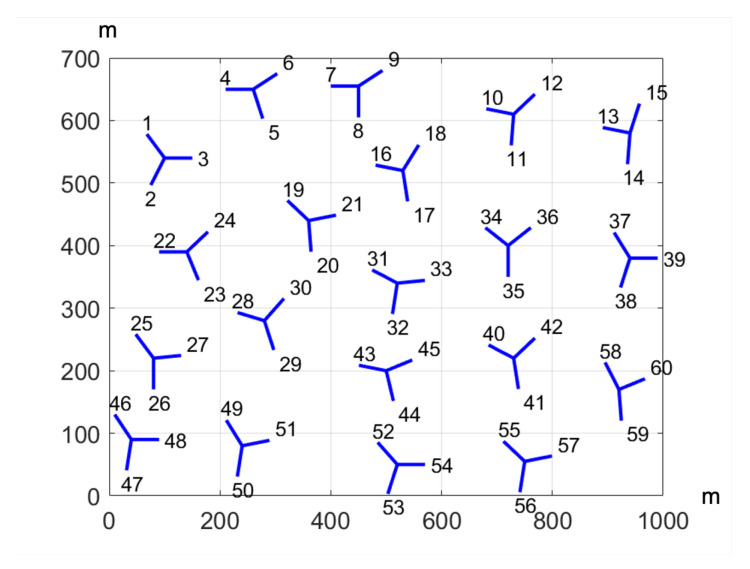
Dense urban scenario considered in this work.

**Figure 3:**
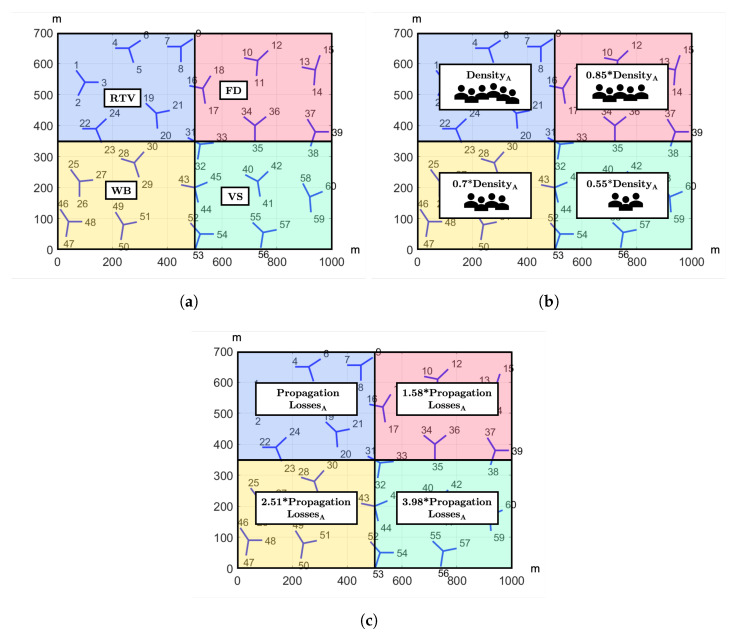
Configurations of the scenario in second, third and fourth tests. (**a**) Second scenario setup. (**b**) Third scenario setup. (**c**) Fourth scenario setup.

The performance of the proposed solution is assessed in a Matlab simulator with MR-DC capabilities, which is based on [34]. The introduced approach is compared with the 3GPP solution based solely on RSRP, so the benefit of the proposed methodology can be highlighted. They are tested in a non-standalone dense urban scenario that correspond to a region of a real celullar network with 20 tri-sectional sites fulfilling 3GPP specification [24]. Figure 2 depicts this scenario. It should be also noted that the eNBs and the gNBs are co-located in each site, so only small-cells are assumed. However, five different scenario setups are proposed to analyze the feasibility of the methodology. In this manner, a uniform distribution of users and services over the whole scenario is set in the first tests, so all regions have equal characteristics. However, the next four scenario setups will consist of four squares of the same size but different characteristics. In particular, an uneven distribution of services is set in the second set of tests. On the other hand, an unequal distribution of users is configured in the third set of tests, resulting in less bandwidth available per UE as density increases. In a similar manner, regions with different level of propagation losses are simulated in the fourth set of tests. Finally, last tests combine the features of the second, third and fourth configurations. Further details of each scenario are collected in Table 1, as well as a graphical representation is shown in Figure 3.

The benefit of the proposed approach is analyzed in terms of MOS reported by users for each of the four services. They are real time video (RTV), video streaming (VS), whose functioning implies a non-real time service, file download (FD) and web browsing (WB) [35]. Their MOS values are computed based on the models introduced in [32]. Regarding FD and WB, it relies on the average session throughput. As for VS service, the MOS depends on the initial buffering time as well as the average stalling frequency and duration. Finally, the MOS of RTV relies on the playout time stored in the receiver buffer. Table 2 summarizes the main simulation parameters.

## 5. Evaluation

**Figure 4:**
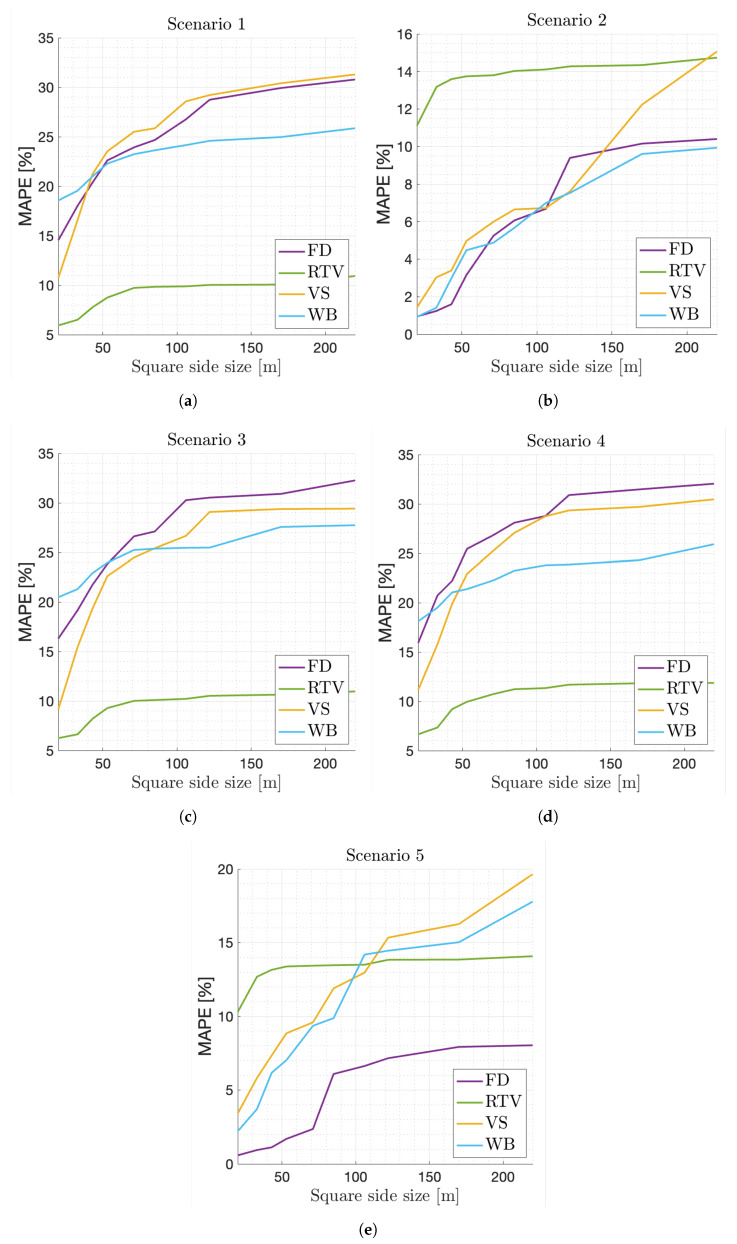
MAPE for different square side sizes, services and scenarios. (**a**) MAPE in Scenario 1. (**b**) MAPE in Scenario 2. (**c**) MAPE in Scenario 3. (**d**) MAPE in Scenario 4. (**e**) MAPE in Scenario 5.

First of all, the optimal square size must be defined. This will indicate the number of estimation models to be stored, as well as the required accuracy in locating the users. With this purpose, the MAPE obtained for each service with different square sizes is computed for all the scenario setups introduced in the previous section. Figure 4 shows that the MAPE decreases for all services as the square side size decreases in every scenario, i.e., the scenario is characterized with more estimation models. However, the values are different for each scenario configuration. The lowest overall MAPE values are obtained in scenarios 2 and 5, as can be seen in the Figure 4b,e. This indicates that the MAPE tends to be lower in small regions where there is a predominant service whose performance is well known. However, the MAPE of the RTV service is higher in these scenarios because the increase in user density causes an increase in the overall delay that negatively affects the QoE perceived by users and is not directly measured by the proposed approach. On the other hand, the MAPE obtained for the other services is similar in each scenario, as they are more dependent on the throughput, which can be better estimated in small regions where local radio conditions and typical resource availability are more accurately known. In conclusion, the lowest MAPE values are obtained for areas with 20 m of square side size. Nonetheless, 30 m of square side size is proposed to generate the estimation models used in the next tests. This decision enables a compromise between the most reliable decisions possible and the minimum memory and computing cost, since the difference between the MAPE obtained for 20 and 30 m of square side size is relatively small. Moreover, this allows for a larger error in tracking the UE location.

**Figure 5:**
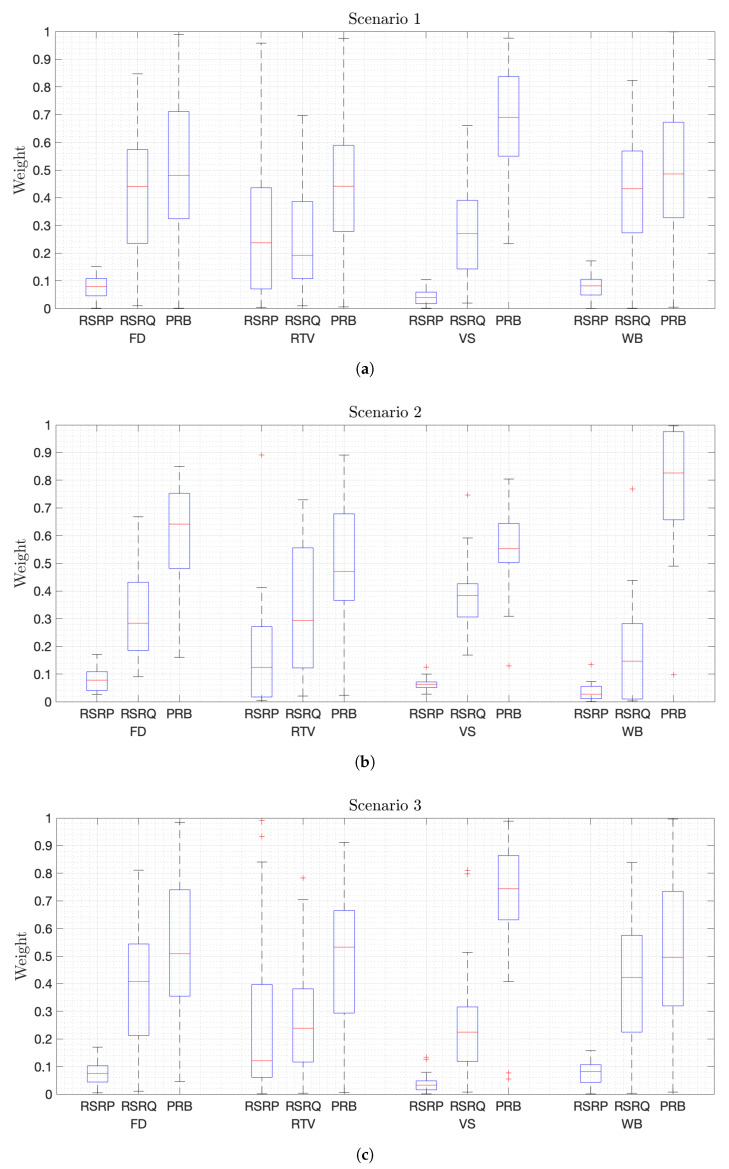

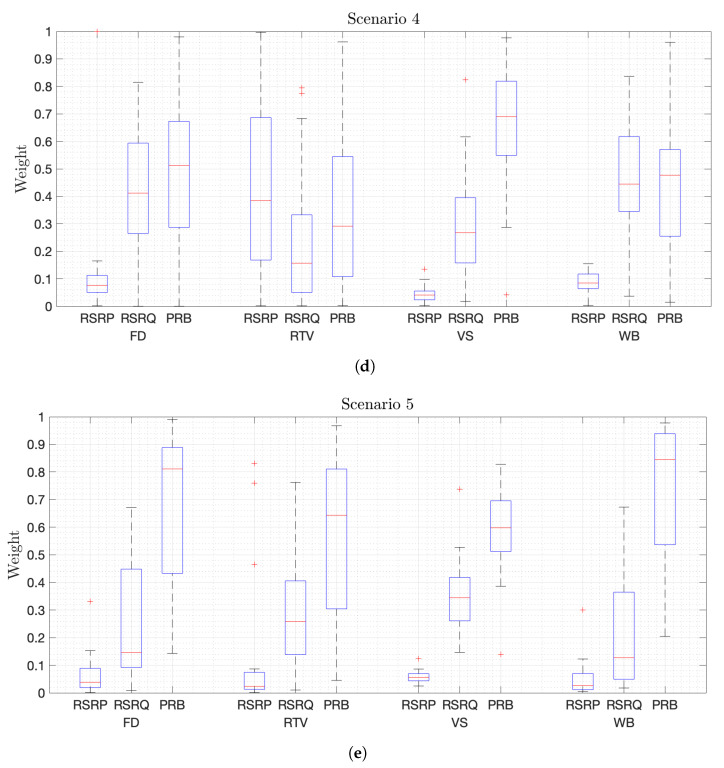
Weight of each metric of the estimation model for each service and scenario. (**a**) Weights in Scenario 1. (**b**) Weights in Scenario 2. (**c**) Weights in Scenario 3. (**d**) Weights in Scenario 4. (**e**) Weights in Scenario 5.

In addition, the importance of each metric in the QoE estimation must be analyzed. In this way, the weight of each metric for each service in all scenarios is represented in a box plot in Figure 5. These values are obtained after normalizing the weight of each metric in each estimation model generated for areas of 30 m of square side size. Figure 5a shows that RSRP is the least important metric for QoE estimation, except for the RTV service since RSRP is correlated with the distance between the nodes and the user, which may have an impact on the service latency and, therefore, on the QoE perceived. On the other hand, RSRQ and available PRBs have similar weights for less resource demanding services, i.e., FD and WB. On the contrary, available PRBs are more important for video services, especially for the most resource and throughput demanding service, i.e., VS. In addition, the relevance of available resources will increase in Scenario 2 for all services, as shows in Figure 5b, since the higher density of video users in two different areas will lead to overload these regions. Moreover, resource availability will be more important for WB users which do not demand as much throughput as FD users, which will also rely heavily on RSRQ. Furthermore, RSRQ is also very relevant for video users to perceive a good QoE. On the other hand, the decrease in user density does not affect the weights much, as the values represented in Figure 5c are similar to those obtained in Scenario 1. Conversely, the increase in propagation losses does affect the RTV service, which is more dependent on RSRP, as depicted in Figure 5d. These additional propagation losses could indicate a longer distance between nodes and users, which is relevant for RTV with strict delay requirements. Finally, Figure 5e shows that available PRBs are considerably more important than the others metrics when there are heavily loaded areas in the scenario, as in Scenario 2. After resource availability, RSRQ is the most important metric. It should also be noted that the relevance of RSRP for RTV users is low in this scenario because there are no additional propagation losses in the area where this service predominates. Therefore, this set of subfigures shows that RSRP, which is the only metric used in the 3GPP solution, is the indicator that has the lowest weight for estimating QoE. This implies that RSRQ and available resources should be used to decide the nodes that will serve an user if MOS is to be maximized. Last but not least, the variance of the values indicates that the weights change throughout the scenario, verifying the requirement of multiple estimation models.

**Figure 6:**
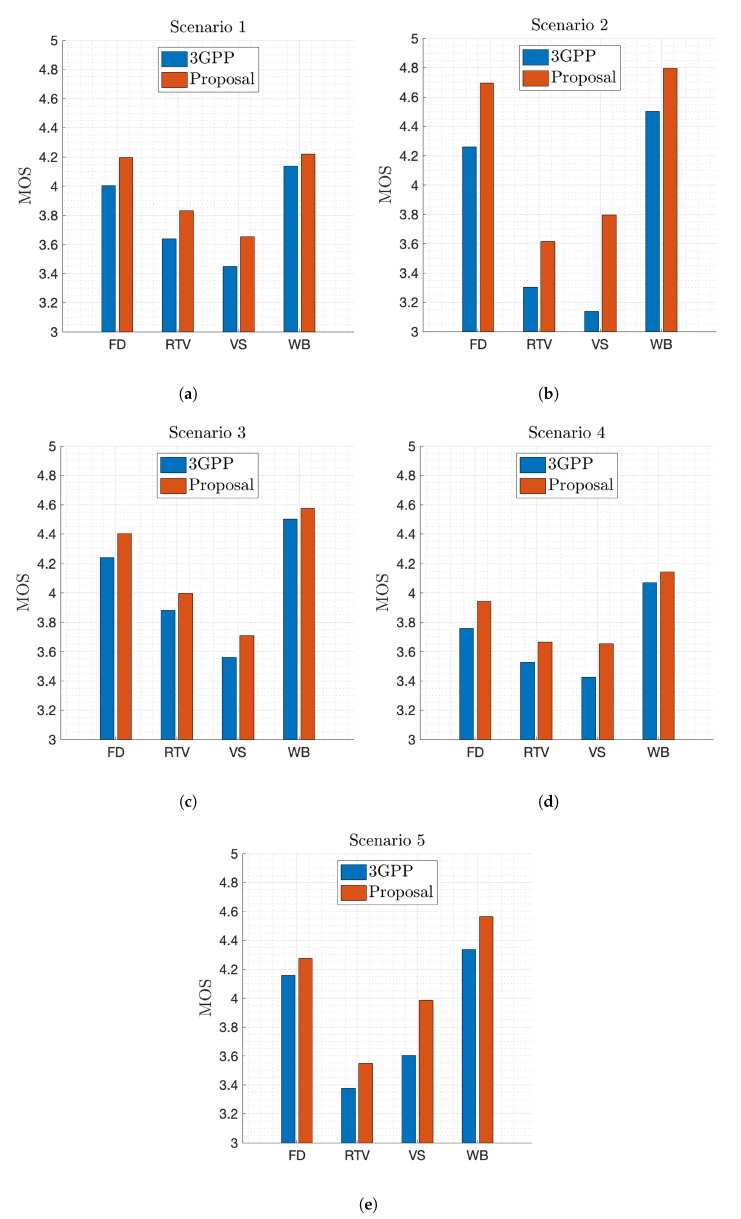
MOS obtained by each solution for each service in every scenario. (**a**) MOS in Scenario 1. (**b**) MOS in Scenario 2. (**c**) MOS in Scenario 3. (**d**) MOS in Scenario 4. (**e**) MOS in Scenario 5.

The previously stored estimation models are now used to select the eNB and gNB that provide the maximum estimated QoE in these tests. The resulting performance is compared with that obtained in the same scenarios but selecting the nodes based solely on RSRP, as indicated by 3GPP. Results show that the proposed solution increases the average MOS obtained by the 3GPP approach for all the services introduced in every scenario, as depicted in Figure 6. Nevertheless, the average MOS achieved varies depending on the scenario configuration. Figure 6b shows how the average MOS of least demanding services increases in the areas where they are predominant with respect to that obtained in the uniform scenario shown in Figure 6a. On the contrary, the MOS of video services decreases due to the high struggle for radio resources in regions where video services are predominant. On the other hand, the MOS of all services increases in Scenario 3 as the user density decreases (Figure 6c). In contrast, the opposite effect is observed when propagation losses grow (Figure 6d). To conclude, Figure 6e shows that the MOS of the predominant services in regions with lower user density is larger despite higher propagation losses. However, the QoE perceived by RTV users is similar to that perceived in Scenario 2, as most RTV users have similar conditions in these two scenarios, as can be seen in Figure 3.

**Figure 7:**
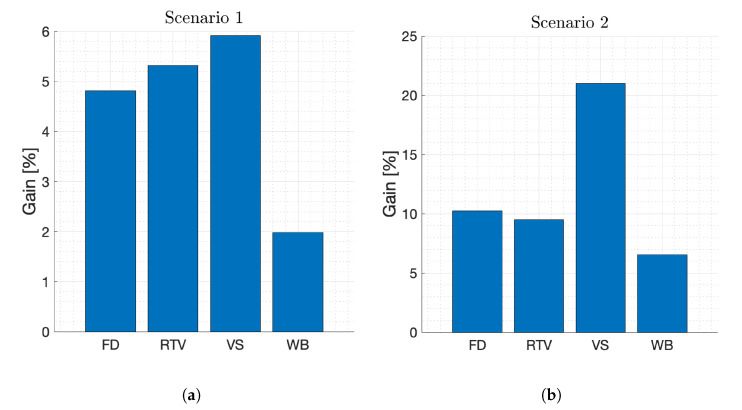

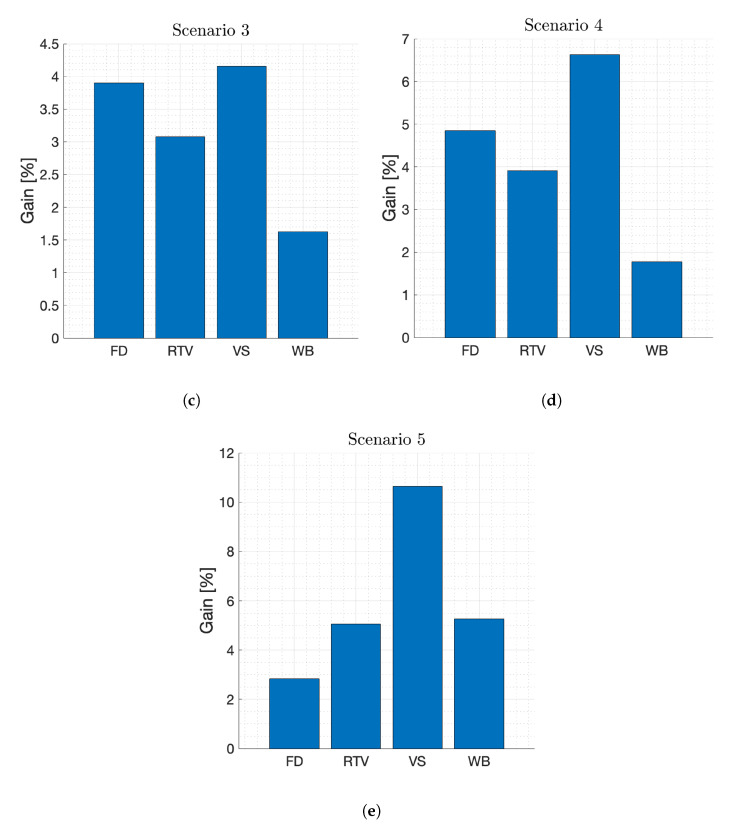
Percentage of gain achieved by the proposed solution for each service in every scenario. (**a**) Gain in Scenario 1. (**b**) Gain in Scenario 2. (**c**) Gain in Scenario 3. (**d**) Gain in Scenario 4. (**e**) Gain in Scenario 5.

To thoroughly analyze the improvement in each service and scenario, Figure 7 represents the percentage of gain of the proposed methodology compared to the 3GPP solution. As can be noted, WB almost always obtains the lowest gain because these users are the least demanding and already perceive a good QoE when using MR-DC, so the MOS is not much affected by the proposed node selection. However, FD users demand more throughput. Therefore, the higher QoE gain indicates that this throughput improvement is more valued by FD users. On the other hand, the MOS of video users is lower than that of FD and WB users in all scenarios (Figure 6), as they demand higher throughput as well as greater availability of radio resources that does not lead to video stalls. In this sense, the MOS of VS users is even lower due to the higher video quality. Hence, the MOS gain obtained by proper node management is more noticeable for this service, especially in scenarios 2 and 5 where there are regions with a large number of VS users struggling to access network resources. To conclude, it is also worth noting that the MOS improvement is more prominent in Scenario 2 (Figure 7b), where different regions of the network usually have a high traffic flow of the same service, making it particularly fruitful to deploy the proposed approach in this type of regions. Therefore, the results demonstrate that the QoE perceived by users of a mobile network can be optimized by an alternative node management in MR-DC-enabled scenarios. To maximize the QoE, the methodology must adapt to the characteristics of the area surrounding the users as well as the service used. In addition, network load and quality metrics must be considered.

## 6. Conclusions and Further Work

The arrival of MR-DC enables great flexibility in the management of network resources, since two nodes are simultaneously connected to a user. Among other advantages, this allows to increase the bandwidth allocated to each user, which can have a positive impact on the QoE. However, a more complex resource management must be tackled. The proposed methodology assigns the user the eNB and gNB that will provide the highest QoE. The results show how this decision varies depending on the characteristics of the area surrounding the user, in addition to the service used. Furthermore, the results demonstrate how resource availability and user-reported RSRQ are more relevant than RSRP in making this selection. In this regard, the performance tests show the improvement of the average QoE compared to the 3GPP solution in a wide variety of scenarios.

The methodology has been tested with different services used by indoor and pedestrian users, but other services involving faster mobility can lead to complications in the dynamic management of connections. Hence, an additional subsystem must be added to address these challenges. This study is left for future work.

## Figures and Tables

**Table 1:** Configuration of the scenario for each test.

**Scenario 1**	Uniform distribution of users and services
	(60 UEs per site and 300 UEs per service on average)
**Scenario 2**	Uneven distribution of services
	4 squares: 70% of total UEs use the same service in each square
	The remaining 30% use the other 3 services
	Predominant service as indicated in Figure 3a
**Scenario 3**	Uneven distribution of users
	4 squares: Users density decreases by 15% in each square
	as indicated in Figure 3b
**Scenario 4**	Different radio conditions
	4 squares: Propagation losses increase in each square
	as indicated in Figure 3c
**Scenario 5**	4 squares with completely different characteristics
	Scenarios 2, 3 and 4 are combined

**Table 2:** Simulation assumptions.

**Environment**	Dense urban scenario with 20 sites,
	1 eNB and 1 gNB per site, 3 sectors/site
**Carrier**	eNB: 10 MHz carrier bandwidth at 2.4 GHz
	gNB: 10 MHz carrier bandwidth at 4 GHz
**Channel model**	Urban micro (UMi) [36]
**PHY numerology**	15 kHz sub-carrier spacing
	12 subcarriers/PRB (180 kHz)
**Data channel MCS**	QPSK to 64 QAM
	with same encoding rates as specified for LTE
**Antenna configuration**	2 × 2 MIMO
**Scheduler**	Classical exponential/proportional fair [37]
**Link adaptation**	CQI-based
**Mobility model**	50% chance of the user being static or moving at 0.83 m/s
**FD traffic model**	File size: log-normal distribution (avg. 2 MB)
**WB traffic model**	Web page size: log-normal distribution (avg. 3 MB)
	No. pages per session: log-normal distribution (avg. 4)
	Reading time: exponential distribution (avg. 30 s)
**RTV traffic model**	Video codec: H.264, Resolution: 720 p
	Video bitrate range: 1.5–2 Mbps
	Video duration: uniform distribution [0, 540] s
**VS traffic model**	Video codec: H.264, Resolution: 1080 p
	Video bitrate range: 3–4 Mbps
	Video duration: uniform distribution [0, 540] s

## Data Availability

Not applicable.

## Abbreviations

CN	Core Network
CSI	Channel State Information
DC	Dual Connectivity
eNB	evolved Node B
FD	File Download
gNB	5G New Radio node B
IoT	Internet-of-Things
KQI	Key Quality Indicator
MAPE	Mean Absolute Percentage Error
MOS	Mean Opinion Score
MR-DC	Multi-Radio Dual Connectivity
PRB	Physical Resource Block
QoE	Quality of Experience
QoS	Quality of Service
RAT	Radio Access Technology
RRC	Radio Resource Control
RSRP	Reference Signal Received Power
RSRQ	Reference Signal Received Quality
RTV	Real Time Video
SINR	Signal-to-Interference-plus-Noise Ratio
UE	User Equipment
UMi	Urban micro
VS	Video Streaming
WAP	Wireless local area network Access Point
WB	Web Browsing
